# Human Cerebrospinal Fluid Induces Neuronal Excitability Changes in Resected Human Neocortical and Hippocampal Brain Slices

**DOI:** 10.3389/fnins.2020.00283

**Published:** 2020-04-21

**Authors:** Jenny Wickham, Andrea Corna, Niklas Schwarz, Betül Uysal, Nikolas Layer, Jürgen B. Honegger, Thomas V. Wuttke, Henner Koch, Günther Zeck

**Affiliations:** ^1^Neurophysics, Natural and Medical Sciences Institute, University of Tübingen, Reutlingen, Germany; ^2^Graduate School of Neural Information Processing/International Max Planck Research School, Tübingen, Germany; ^3^Institute for Ophthalmic Research, University of Tübingen, Tübingen, Germany; ^4^Department of Neurology and Epileptology, Hertie Institute for Clinical Brain Research, University of Tübingen, Tübingen, Germany; ^5^Department of Neurosurgery, University of Tübingen, Tübingen, Germany; ^6^Department of Epileptology, Neurology, RWTH Aachen University, Aachen, Germany

**Keywords:** human tissue, human cerebrospinal fluid, microelectrode array, CMOS-MEA, hippocampus, cortex, organotypic slices

## Abstract

Human cerebrospinal fluid (hCSF) has proven advantageous over conventional medium for culturing both rodent and human brain tissue. In addition, increased activity and synchrony, closer to the dynamic states exclusively recorded *in vivo*, were reported in rodent slices and cell cultures switching from artificial cerebrospinal fluid (aCSF) to hCSF. This indicates that hCSF possesses properties that are not matched by the aCSF, which is generally used for most electrophysiological recordings. To evaluate the possible significance of using hCSF as an electrophysiological recording medium, also for human brain tissue, we compared the network and single-cell firing properties of human brain slice cultures during perfusion with hCSF and aCSF. For measuring the overall activity from a majority of neurons within neocortical and hippocampal human slices, we used a microelectrode array (MEA) recording technique with 252 electrodes covering an area of 3.2 × 3.2 mm^2^. A second CMOS-based MEA with 4225 sensors on a 2 × 2 mm^2^ area was used for detailed mapping of action potential waveforms and cell identification. We found that hCSF increased the number of active electrodes and neurons and the firing rate of the neurons in the slices and induced an increase in the numbers of single channel and population bursts. Interestingly, not only an increase in the overall activity in the slices was observed, but a reconfiguration of the network could also be detected with specific activation and inactivation of subpopulations of neuronal ensembles. In conclusion, hCSF is an important component to consider for future human brain slice studies, especially for experiments designed to mimic parts of physiology and disease observed *in vivo*.

## Introduction

The brain, with all its neurons, glial cells, and blood vessels, is cushioned and protected by a colorless fluid called cerebrospinal fluid (CSF). CSF can be found in the space between the brain surface and the skull, the subarachnoid space, in all the ventricles as well as in and around the spinal cord. CSF is a fluid characterized by a low percentage of protein and high percentage of salts and is in constant contact with the interstitial fluid of the parenchyma, the fluid located between neurons and glial cells all over the brain. The close connection with the interstitial fluid gives the CSF a potential to act as a vessel for different neuromodulatory signals (Agnati et al., 1986, 2010) and through these signals to modulate neuronal excitability and activity. “Activity levels” is used throughout this manuscript as a substitute term characterizing functional changes not only at the single cell level but also at the cell population level upon changing the extracellular recording solution. Experimental findings summarized in the review by Bjorefeldt et al. (2018) showed that human CSF (hCSF) can influence the function of neurons in both hippocampal slices and cortical neuronal cultures from rat. In general, all electrophysiological experiments performed, using either rodent brain or human brain slices, normally are performed using a solution called artificial CSF (aCSF) for perfusion of the tissue during the experiments. The aCSF is carefully made with several different salts together with glucose to mimic the components found in hCSF. But hCSF also contains a number of neuromodulators such as neurotransmitters, neuropeptides, neurosteroids, purines, and endocannabinoids, which are not present in aCSF (Skipor and Thiery, 2008). When hCSF was compared to aCSF in experiments with rat slices, the spontaneous firing of action potentials increased in both hippocampal and cortical slices (Bjorefeldt et al., 2015). This activity seemed closer to the highly active states observed during recordings *in vivo.* The activation of G-protein coupled receptors are postulated to be the reason for the enhanced activity. In addition, clear advantages utilizing hCSF as the culturing medium for human organotypic brain slices have previously been shown by our group (Schwarz et al., 2017, 2019). These cultures, acquired from resected human brain tissue, proved more viable and functionally intact over both short and long incubation times when using hCSF compared to conventional culturing medium (Schwarz et al., 2017). Using resected human brain tissue as an *ex vivo* platform for modeling diseases or to study healthy brain activity provides huge translational advantages. Both better understanding of the human physiology and disease mechanisms have been investigated with this *ex vivo* system (Wittner et al., 2001; Sandow et al., 2015; Dossi et al., 2018; Mansvelder et al., 2019).

As stated above, changes in the activity of neuronal networks were described for rodent tissue and cells, but if hCSF affects the function of human neurons and modulates the level of activity is still an open question. The aim of our present study is to investigate whether perfusion with hCSF could modulate the neuronal activity differently, on the single cell and network level, compared to perfusion with the commonly used aCSF. We hypothesized that, just as was seen in the rodent studies, the firing activity of neurons will increase with perfusion of hCSF compared to aCSF. We performed extracellular recordings using high-density microelectrode arrays (MEAs) to study both the response from individual neurons and how their activity contributes to the overall network activity. *Ex vivo* measurements of single cells and small networks open interesting avenues for research, but the translation of the results back to the *in vivo* state remains challenging. It is therefore of great importance to design *ex vivo* experiments mimicking the *in vivo* conditions as closely as possible. Usage of hCSF, as the recording solution, might be therefore an interesting alternative option for specific experimental questions.

## Materials and Methods

### Human Slice Preparation and Culture

**FIGURE 1:**
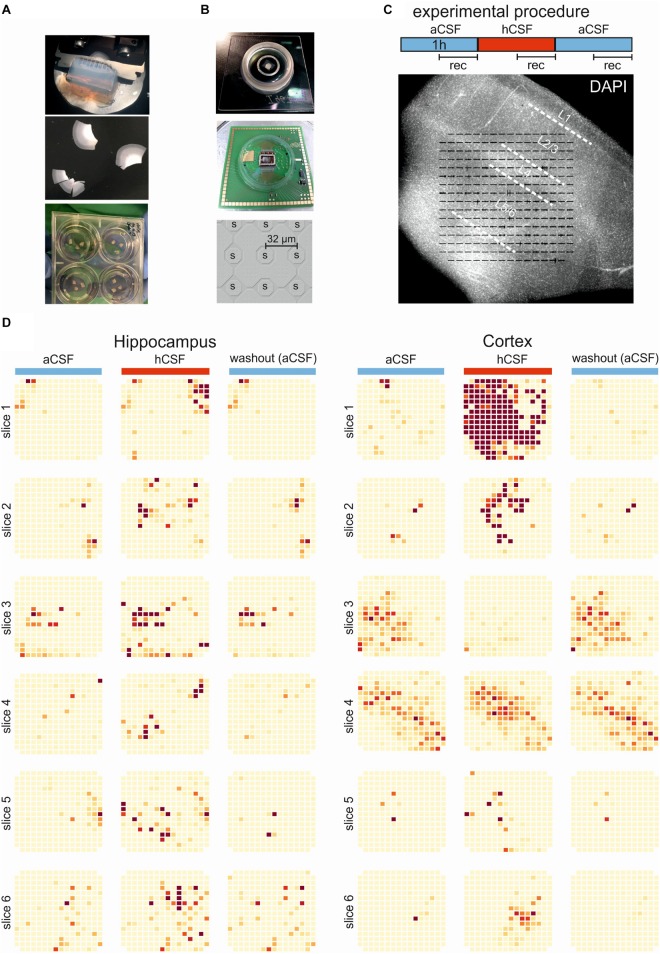
Experimental and overview of activity. **(A)** The brain tissue is surgically resected *en bloc* and the tissue block (top) is cut into 250-μm slices (middle) using a vibratome. The slices are incubated on membrane insets in six-well plates (bottom) with hCSF as medium for up to 2 weeks. **(B)** Pictures of the 256-MEA chip (top), the CMOS-MEA chip (middle), and the spacing of the CMOS-MEA electrodes (bottom). **(C)** Experimental protocol. Slices were perfused with aCSF for an acclimation period of 30 min, followed by 60-min aCSF (blue bar), 60-min hCSF (red bar), and again 60-min aCSF (blue bar) perfusion. Recording was performed continuously for 30 min in aCSF, hCSF, and aCSF, as indicated. Underneath, a typical human slice culture is shown with all cortical layers (L1–L6) and overlay of the MEA on the slice. **(D)** Qualitative overview over all 12 slices recorded using 256-MEA. When hCSF was washed in, the general activity of slices increased, with new active areas emerging and with the already active areas showing a further increase in number of spikes presented here as heat maps. Each electrode is represented by a small square and darker color indicates higher activity (spike rate). Activity was normalized for each slice to the maximum spike rate per electrode in aCSF.

Human hippocampal and cortical organotypic slice cultures were prepared from resected tissue obtained from patients undergoing epilepsy surgery. For this study, we collected tissue and included data of six patients. All patients were surgically treated for intractable epilepsy. Approval (# 338/2016A) from the ethics committee of the University of Tübingen together with written informed consent from all patients allowed spare tissue from resective surgery to be included in our study. Hippocampus and cortex were carefully micro-dissected and independently resected *en bloc* to ensure tissue integrity, directly transferred into ice-cold aCSF (in mM: 110 choline chloride, 26 NaHCO_3_, 10 D-glucose, 11.6 Na-ascorbate, 7 MgCl_2_, 3.1 Na-pyruvate, 2.5 KCl, 1.25 NaH_2_PO_4_, and 0.5 CaCl_2_) equilibrated with carbogen (95% O_2_, 5% CO_2_), and immediately transported to the laboratory. Tissue was kept submerged in cool and carbogenated aCSF at all times. For hippocampal slices, the tissue was trimmed to give a flat glue surface in the coronal plane, glued onto the slicing platform, and then sliced into 250-μm-thick slices using a Microm HM 650 V vibratome (Thermo Fisher Scientific Inc.). Cortical slices were prepared as follows. After removal of the pia, tissue chunks were trimmed perpendicular to the cortical surface and 250–350-μm-thick acute slices were prepared using a Microm HM 650 V vibratome (Thermo Fisher Scientific Inc.). Subsequently, the slices were transferred onto culture membranes (uncoated 30 mm Millicell-CM tissue culture inserts with 0.4 μm pores, Millipore) and kept in six-well culture dishes (BD Biosciences) (Figure 1A). HCSF was used as culture medium (1.5 mL per insert), as described in previous studies and was exchanged every 2–3 days (Schwarz et al., 2017, 2019). The plates were stored in an incubator (Thermo Scientific) at 37°C, 5% CO_2_, and 100% humidity. Slices for patch-clamp recordings were carefully removed from the culture insert membrane with a brush and then transferred into the recording chamber. For MEA recordings, the culture insert membrane was cut around the slice with the slice still attached to the membrane. With forceps, the membrane, with slice attached, was moved to the MEA-chip and placed with the slice surface facing down onto the electrodes. Slices were allowed at least 30 min to equilibrate before patch-clamp or MEA recordings were started. Only slice cultures with a minimum of two viable slices were analyzed after the MEA recording. HCSF was collected from patients with suspected normal pressure hydrocephalus (NPH). We received and pooled hCSF of several patients who needed to undergo a tap test either by lumbar puncture or lumbar drain as part of the diagnostic workup for NPH. It is well established and known from daily clinical practice that hCSF of NPH patients exhibits physiological hCSF parameters (lactate, glucose, cell count, and protein levels; Schwarz et al., 2017), indistinguishable from the ones of healthy individuals (Bjorefeldt et al., 2015). The hCSF was centrifuged at 4000 r/min at 4°C for 10 min and the supernatant was collected and stored at −80°C.

### MEA Recordings

High-density MEAs have been used recently to study single-cell activity as well as population activity in rodent brain slices (Ferrea et al., 2012; Gong et al., 2016; Zeck et al., 2017). Here we used two MEA types, each of them with specific characteristics and advantages. A 256–MEA (16 × 16 lattice) with electrode size 30 μm and electrode spacing of 200 μm thus covers a recording area of ∼3.2 × 3.2 mm^2^ (USB-MEA 256-System, Multi Channel Systems MCS GmbH, Figure 1B). The second MEA was a complementary metal oxide semiconductor-based MEA (CMOS-MEA32) used with a commercial setup (CMOS-MEA5000-System, Multi Channel Systems MCS GmbH). The CMOS-MEA comprised 4225 sensor sites (65 × 65 sensor lattice) with an electrode pitch of 32 μm, thus covering a recording area of ∼2 × 2 mm^2^ (Figure 1B). Recordings with the 256-MEA were performed at a sampling rate of 25 kHz using the MC Rack software (Multi Channel Systems MCS GmbH) while recordings with the CMOS-MEA were performed at 20 kHz using CMOS-MEA Control software (Multi Channel Systems MCS GmbH, version 2.2.0). Seven hippocampal slices from two hippocampectomy procedures and eight cortical slices from three cortical resections were collected for extracellular recording using 256-MEAs (six hippocampal slices, six cortical slices) and CMOS-MEA (one hippocampal slice and two cortical slices). After 7–18 days *in vitro* in hCSF, the slices were placed in a MEA recording chamber, perfused with aCSF, containing (in mM) 118 NaCl, 3 KCl, 1.5 CaCl_2_, 1 MgCl_2_, 25 NaHCO_3_, 1 NaH_2_PO_4_, 30 D-glucose, and equilibrated with carbogen (95% O_2_ and 5% CO_2_, pH 7.4) and heated to 34°C for an acclimation period of 30 min. After acclimation, aCSF was perfused for an additional hour (we name this period *aCSF*) followed by 1 h of hCSF perfusion (we name this period *hCSF*) and then a final hour with aCSF (we name this period *washout*) (Figure 1C). To avoid analyzing data from the transition time from one medium to the next, the analysis window was defined as the last half hour of each condition (Figure 1C). In four of the slices, an extra condition was added at the end, aCSF with high potassium (high K aCSF) concentrations. For this “high K aCSF solution,” potassium chloride was added to the aCSF, resulting in a potassium concentration of 6–8 mM. In addition, three slices were recorded in hCSF (60 min perfusion with the last 30 min being recorded) followed by recording in high K aCSF (60 min perfusion with the last 30 min being recorded).

### Patch-Clamp Recordings

Slices were positioned in a submerged-type recording chamber (Scientifica, United Kingdom/Warner apparatus), continuously superfused with aCSF and visualized with a BX61WI Microscope (Olympus) or a Leica stereomicroscope. Recordings were performed using recording electrodes with a resistance of 3–5 MΩ and filled with an intracellular whole-cell patch-clamp pipette solution containing the following components (in mM): 140 K-gluconic acid, 1 CaCl_2_^∗^6H_2_O, 10 EGTA, 2 MgCl_2_^∗^6H_2_O, 4 Na_2_ATP, and 10 HEPES, pH 7.2, 300 mOsm. The intracellular whole-cell patch-clamp pipette solution contained biocytin (5 mg/mL) to allow for *post hoc* identification of the location and morphology of recorded neurons. Whole-cell current-clamp recordings were obtained from cortical neurons using either the visual-patch or blind patch technique and sampled at 20 or 100 kHz with a low-pass filter of 5 or 30 kHz. Recordings were performed with unpolished patch electrodes manufactured from borosilicate glass pipettes with filament (Science products). Patch-clamp experiments were performed with a patch-clamp amplifier (Multiclamp 200B) or an NPI Bridge Amplifier (Model BA-01X), a digitizing interface (Digidata 1440A or 1550A Digidata), and pClamp 10 software (Molecular Devices). The junction potential was calculated and subtracted offline to correct the membrane potential in current clamp mode.

### Data Analysis and Statistics

The analysis of recordings from 256-MEAs was performed using Python (version 3.6, including the libraries Numpy, Scipy). Recordings were bandpass-filtered (150–5000 Hz, Butterworth second order) and spikes were detected as described (Quiroga et al., 2004) using a 1-ms post-spike dead time. The firing rate for each electrode was calculated based on the number of threshold crossings within the recording time (30 min). To reduce the number of false-positive detection of active electrodes, only electrodes with more than 90 identified spikes in the 30-min-long recording were considered as active. Thus, the average spike rate per slice comprised only electrodes with a spike rate higher than 0.05 Hz. The color scale of the heat maps displaying the average firing rate in each slice (Figure 1D) was normalized to the maximum firing rate detected in aCSF (first condition) for each slice. We mention that the threshold crossings evaluated further as spikes may represent multi-unit activity, i.e., signals detected from several cells. It has not been determined here how many neurons contribute to the activity on one electrode; therefore, “spike rate” should be understood as a multi-unit activity within the specified time interval.

Single electrode bursts were defined as having at least three consecutive spikes with a maximum of 100-ms inter-spike interval. This definition was adapted from a previous report (Chen et al., 2009) and the joint inter-spike interval histogram of individual electrodes. Increasing or decreasing firing rate between aCSF and hCSF was inferred if the relative change exceeded 50%. Electrodes with less than 50% activity change were considered stable.

The population bursts were defined as described previously (Theiss et al., 2017; Koch et al., 2019). In brief, for the identification of population bursts, all detected spikes were merged in consecutive non-overlapping 5-ms bins. This raw population firing rate was smoothed using a normalized Gaussian kernel with standard deviation 100 ms. Network burst onset was determined when this smoothed population firing rate exceeded the slowly varying one-second moving average. Very low-firing population bursts were discarded, if their peak firing rate did not exceed 10% of the average of the top five peaks. Population burst detection was also checked by visual inspection to avoid detection of occasional artifacts present on all electrodes.

In addition to the absolute parameter values (firing rate, population burst rate), we calculated the relative change when switching from recording solution A to recording solution B. The relative change was calculated as the (parameter _*hCSF*_ - parameter _*aCSF*_)/(parameter _*hCSF*_ + parameter _*aCSF*_). This ratio is 1 if cells are active only in hCSF and -1 if cells are activity only in aCSF.

**FIGURE 2:**
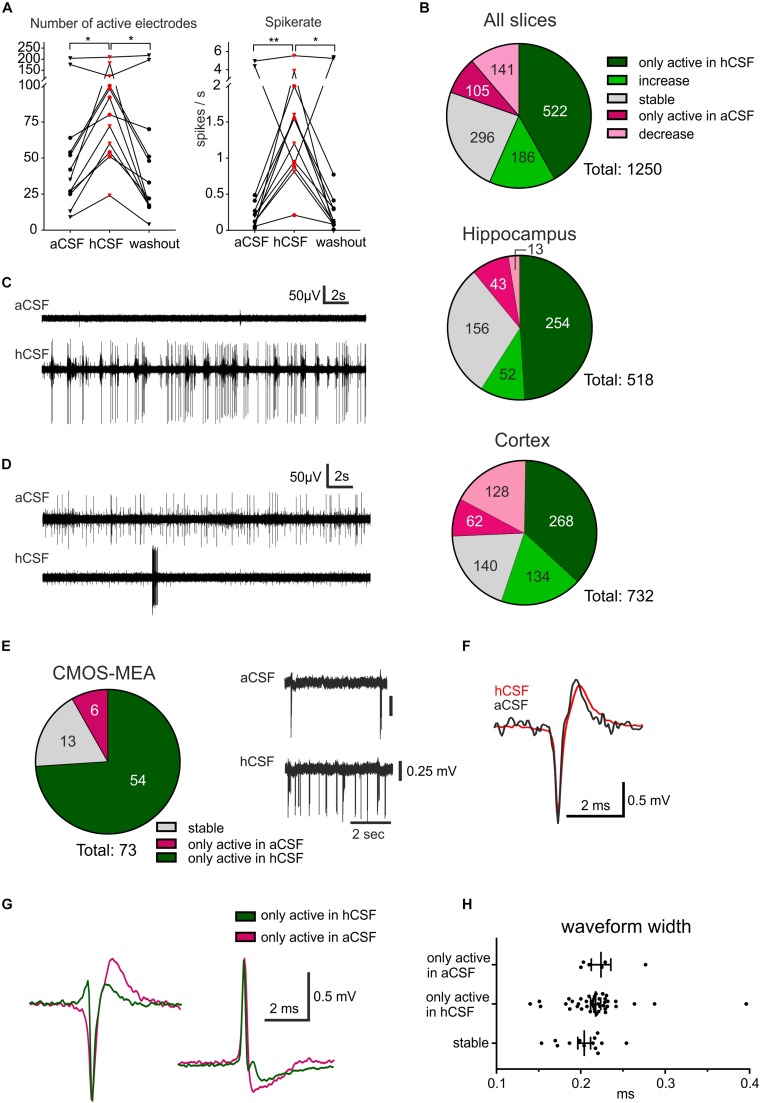
hCSF induces a general increase in neuronal activity and specific activation and inactivation of subpopulations of neurons. **(A)** The general activity measured by the number of active electrodes and spike rate, with an average of each hippocampus slice represented by a circle and each cortex slice as a triangle, increased when hCSF was washed in and then decreased again when hCSF was washed out. The average number of electrodes and spike rate in one cortical slice did not increase but decreased instead, this can also be seen in the corresponding heat map in Figure 1D (cortex slice 3). **(B)** Overview presenting identified electrodes recording activity only in hCSF (dark green), electrodes recording increased spike rate in hCSF (green), recording a decreased spike rate in hCSF (light red), electrodes only active in aCSF (red), and electrodes recording stable activity (for which spike rate increased or decreased by less than 50%). The majority of the electrodes only recorded spikes in hCSF or measured an increased spike rate in hCSF (522 and 186 electrodes, respectively, of a total of 1250 electrodes). Interestingly, 105 electrodes only recorded activity in aCSF and 141 electrodes recorded a decreased spike rate in hCSF compared to aCSF. Examples of electrode recording revealing **(C)** increased activity and **(D)** decreased activity when hCSF was washed in. Changed excitability was also detected in extracellular recordings using the CMOS-MEA. **(E)** Overview of identified single-unit activity (*n* = 73 cells in three slices) with extracellular voltage recorded by a selected sensor showing the increased excitability of one cell in hCSF compared to aCSF. Selected extracellular waveforms of cells active in **(F)** both conditions and **(G)** in hCSF or in aCSF only. **(H)** The waveform width was not different between the spikes recorded from neurons only active in aCSF, only active in hCSF, or active in both (stable); significance is indicated by *p < 0.05; **p < 0.01.

Analysis of the recordings using the CMOS-MEA was performed using CMOS MEA Tools, a software implementing the cICA algorithm (Leibig et al., 2016). Only well-isolated single units were considered (criterium: IsoBG > 5). Due to computational constrains, continuous recordings with the CMOS-MEA were, in the present study, limited to 2-min-long files. The average extracellular waveform of each cell on multiple neighboring electrodes was obtained based on spike sorting. We selected for each cell the electrode with the maximal amplitude and compared the extracellular waveforms during aCSF and hCSF perfusion (Figures 2F–H).

Data are reported as mean ± SEM and statistical significance was evaluated with the non-parametric Friedman test followed by Dunn’s multiple comparison test. Statistical testing was done with Prism 7.01 (GraphPad) and differences between groups were considered significant when *p* < 0.05.

## Results

### Human CSF Induce a General Increase in Neuronal Activity

To quantify the activity level of the 12 slices on the 256-MEAs, we first extracted the number of active electrodes for each condition, showing 60.67 ± 18.15 electrodes active during *aCSF*, 95.42 ± 15.58 during *hCSF*, and 59.08 ± 20.69 during *washout*, which was significantly different between the groups (*p* = 0.0062). In Dunn’s multiple comparison test, we found that the increase in number of active electrodes between *aCSF* and *hCSF* (*p* = 0.0239) and the decrease in number of active electrodes between *hCSF* and *washout* (*p* = 0.0128) were both significantly different, while the number of active electrodes was not significantly different between *aCSF* and *washout* (*p* > 0.9999), altogether demonstrating reversibility of *hCSF*-mediated activity increase (Figure 2A).

We next analyzed the overall spike rate in each slice and found the average spike rate to be 0.95 ± 0.50 spikes/s during *aCSF*, 1.76 ± 0.43 spikes/s during *hCSF*, and 1.08 ± 0.57 spikes/s during *washout*. The average spike rate in each slice increased when washing in hCSF and decreased again when switching back to *aCSF* during *washout*. The data from the three conditions were tested with the paired, non-parametric Friedman test, and the difference was found to be significant (*p* = 0.0014). Dunn’s multiple comparison test found the increase in firing rate when comparing *aCSF* and *hCSF* to be significant (*p* = 0.0016) as well as the decrease between the *hCSF* and the *washout* (*p* = 0.0239) while the spike rate remained stable between *aCSF* and *washout* (*p* > 0.9999) (Figure 2A). In addition, we compared the relative change in average firing rate in hippocampal slices and cortical slices separately (see section “Materials and Methods” for definition of relative change). We found for both preparations an increase in firing rate when changing from aCSF to hCSF (64 ± 7%, hippocampus, 46 ± 25%, cortex) and decreased average activity during washout condition (57 ± 13% in hippocampal slices, 50 ± 20% in cortical slices).

Next, we performed a subgroup analysis and assessed the number of active electrodes and the spike rates separately for hippocampal and cortical recordings. In the hippocampus subgroup, we found the same significant differences as described for the pooled data from both groups, but no significant differences were found in the cortex subgroup (Table 1 for mean ± SEM and *p*-values). This discrepancy is explained by data obtained from one cortical slice, which was clearly going against the trend, together with the relatively low number of slices (*n* = 6). This single slice showed a decrease of number of active electrodes and of spike rate when washing in hCSF (Figure 2A). To better understand why a small subset of the slices displayed such a clear deviation from the rest of the group, we performed a deeper analysis of the activity for each electrode.

**TABLE 1:** Mean and standard error of the mean (SEM) for parameters calculated for the 12 slices recorded in aCSF, hCSF, and washout.

	Mean ± SEM			Friedman test	Dunn’s multiple comparison test, *p*-value		
Active channels	aCSF	hCSF	washout	*p* value	aCSF vs hCSF	hCSF vs washout	aCSF vs washout
All	60.667 ± 18.146	95.417 ± 15.577	59.083 ± 20.694	0.0062	0.0239	0.0128	>0.9999
Hippocampus	44.00 ± 6.372	79.167 ± 8.909	40.00 ± 8.234	0.0055	0.0628	0.0117	>0.9999
Cortex	77.33 ± 36.02	111.67 ± 29.71	78.17 ± 40.87	0.4297	0.4467	0.7446	>0.9999

**Spikes/s**	**aCSF**	**hCSF**	**washout**	***p* value**	**aCSF vs hCSF**	**hCSF vs washout**	**aCSF vs washout**

All	0.945 ± 0.505	1.755 ± 0.432	1.075 ± 0.575	0.0014	0.0016	0.0239	>0.9999
Hippocampus	0.2605 ± 0.068	1.1880 ± 0.259	0.330 ± 0.102	0.0017	0.0045	0.1299	0.7446
Cortex	1.630 ± 0.964	2.323 ± 0.791	1.820 ± 1.105	0.1840	0.2498	0.2498	>0.9999

**Number of bursts**	**aCSF**	**hCSF**	**washout**	***p* value**	**aCSF vs hCSF**	**hCSF vs washout**	**aCSF vs washout**

All	11,637 ± 7616	16,597 ± 9560	12,258 ± 7839	0.0052	0.0322	0.0092	>0.9999
Hippocampus	614.7 ± 242.7	3457.7 ± 1230.2	997.8 ± 450.5	0.0275	0.0424	0.1818	>0.9999
Cortex	22,660 ± 14,373	29,736 ± 18,210	23,518 ± 14,814	0.0695	0.7446	0.0628	0.7446

**Burst duration (s)**	**aCSF**	**hCSF**	**washout**	***p* value**	**aCSF vs hCSF**	**hCSF vs washout**	**aCSF vs washout**

All	0.428 ± 0.058	0.643 ± 0.204	0.385 ± 0.069	0.5580	>0.9999	>0.9999	0.9223
Hippocampus	0.447 ± 0.064	0.968 ± 0.368	0.462 ± 0.097	0.4297	0.7446	0.4467	>0.9999
Cortex	0.409 ± 0.104	0.319 ± 0.079	0.307 ± 0.097	0.4297	0.4467	>0.9999	0.7446

### Specific Activation and Inactivation in Subpopulations of Neurons

Further analysis of the firing rate recorded via each individual electrode detected three distinct responses to washing in hCSF: (i) decreased firing rate, (ii) increased firing rate, and (iii) no change in firing rate. Via an average of 9.3 ± 1.86 electrodes, a decreased firing rate in each slice was detected and through 5.2 ± 1.35 of the electrodes per slice, no spikes during *hCSF* were detected. The number of electrodes through which an increase in spike rate was detected was on average 58.7 ± 8.88 per slice and via 40.3 ± 4.81 of the electrodes, no spikes were detected during *aCSF*. On average, via 11.3 ± 2.60 electrodes, no change (increase or decrease) was detected. These data indicate that individual neurons or subnetworks in the slices respond differently when hCSF is washed in, with some neurons decreasing action potential firing (decreased activity detected via 9.3 ± 1.86 electrodes), but with the majority of neurons responding with increased action potential firing (increased activity detected via 58.7 ± 8.88 electrodes per slice) (Figures 2B–D).

For three additional slices (two from cortex, one hippocampus), we performed a detailed analysis of the specific activation based on identified cellular activity using CMOS-MEAs. Recordings were performed under similar experimental conditions as with the 256-MEAs (Figure 1C, aCSF, hCSF, aCSF). In these recordings, we identified 73 individual cells (see section “Materials and Methods” for spike sorting), while many sensors recording multi-unit activity were removed from further analysis. The majority of identified cells (74%) was only active in hCSF, a small number (8%) was only active in aCSF and the remaining cells (18%) were identified as active in both hCSF and aCSF (Figure 2E). These results support our 256-MEA findings that different subpopulations of neurons may respond distinctly to hCSF and aCSF. For cells identified as active in both aCSF and hCSF (*n* = 13), no change in the waveform could be observed between *aCSF* and *hCSF* (Figure 2F). However, when comparing the waveforms between cells, the shapes were different, with most of them displaying a negative peak and some a positive peak or biphasic (Gold et al., 2009). The average width at half waveform peak was 0.21 ± 0.03 ms with all identified cells included and did not differ between cells only active in aCSF (0.22 ± 0.03 ms), cells only active in hCSF (0.22 ± 0.04 ms), or cells active in both aCSF and hCSF (0.20 ± 0.03 ms) (Figures 2G,H). We identified five cells active in hCSF, which displayed a narrow waveform (<0.16 ms), a potential indication for inhibitory neurons (Viskontas et al., 2007). In summary, the extracellular waveforms did not provide indications for differential effects of hCSF on neurons within human brain slice cultures.

To confirm the preferential activation (increased excitability) of cells by hCSF, we performed whole-cell patch-clamp experiments in current clamp in a small sample of human cortical neurons (*n* = 6, Supplementary Figure S1C). In five out of six cells recorded, spiking increased substantially upon hCSF perfusion compared to aCSF while one cell did not produce action potentials in aCSF or hCSF (Supplementary Figure S1A). This increase was combined with a significant depolarization of the recorded cells from −73.90 ± 3.19 to −67.33 ± 3.05 mV (Supplementary Figure S1B, ^∗^*p* < 0.05), which was reversible by a washout (Supplementary Figures S1A,B).

### Human CSF Induces an Increase in Single-Channel and Population Burst Activity

**FIGURE 3:**
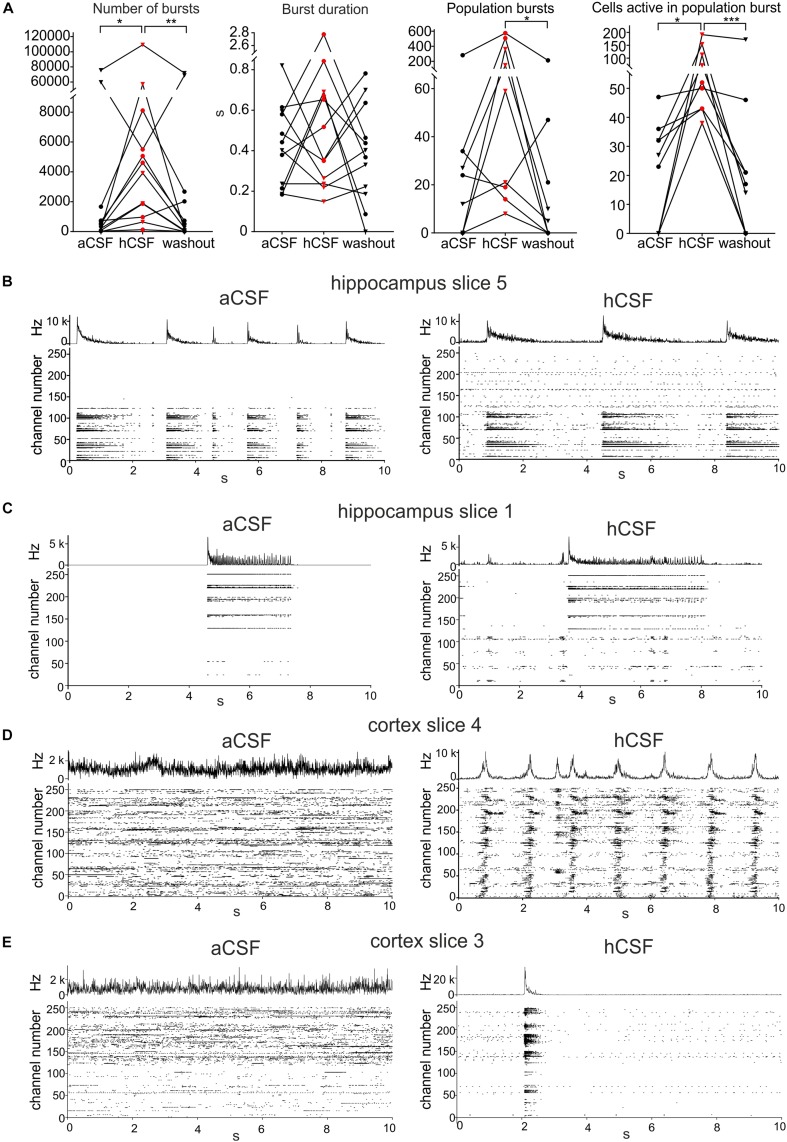
Human CSF promotes synchronized activity. **(A)** The number of bursts detected in single electrodes increased when hCSF was washed in and decreased back toward *aCSF* baseline levels when hCSF was washed out again (each hippocampus slice represented by a circle and each cortex slice as a triangle). Burst duration did not change when hCSF was washed in. The number of population bursts increased in all cortical slices (triangles) and in two of the hippocampal slices (circles) following hCSF wash in and then decreased in all slices but one (hippocampal) during the washout. The number of cells (measured by number of active electrodes) participating in population bursts increased in all slices during hCSF and then decreased back toward baseline levels during washout. Significance is indicated by ^∗^*p* < 0.05; ^∗∗^*p* < 0.01; ^∗∗∗^*p* < 0.001. **(B–D)** Representation of different activity patterns recorded in aCSF (left) and hCSF (right) **(B)** Activity pattern from hippocampus slice 5 (see heat-map in Figure 1D). Upper panels: Summarized spike rate trace. Lower panels: Raster plots showing the activity on all 252 recording channels. Comparison of the raster plots (aCSF vs hCSF) reveals additional active channels in hCSF, which record continuous but not bursting activity. **(C)** Activity patterns from hippocampus slice 1 (heat-map in Figure 1D). Comparison of the raster plots reveals additional channels in hCSF, which record bursting activity in a new area of the slice (channel number 1–100). **(D)** Activity patterns from cortex slice 4 (heat-map in Figure 1D). Comparison of the raster plots shows how non-synchronized spiking activity recorded in aCSF becomes highly synchronized in hCSF across the recorded slice area. **(E)** Activity patterns from cortex slice 3 (heat-map in Figure 1D). Comparison of the raster plots shows a synchronization shift in hCSF with most spikes recorded during the population burst.

To further understand how the neurons changed their activity when hCSF was washed in, we looked specifically at burst activity (defined in section “Materials and Methods”) on individual active MEA electrodes. We found that the average number of bursts in the slices during *aCSF* was 11,637 ± 7616 (evaluated in the 30-min-long recording) and that it was 16,597 ± 9561 during *hCSF*, and 12,258 ± 7839 during the *washout*. These values were significantly different between the groups (*p* = 0.0052). In Dunn’s multiple comparison test, the number of bursts recorded in *aCSF* was significantly lower compared to *hCSF* (*p* = 0.0322). The decrease between *hCSF* and the *washout* was also statistically significant (*p* = 0.0092), while the number of bursts was not significantly different between *aCSF* and *washout* (*p* > 0.9999) (Figure 3A). The duration of the detected bursts was, on average during *aCSF* 0.43 ± 0.06 s, during *hCSF* 0.64 ± 0.20 s, and during *washout* 0.39 ± 0.07 s, and no significant difference was found (*p* = 0.5580) (Figure 3A).

To evaluate the effect hCSF has on synchronous network activity, we continued by detecting the number of population bursts (defined in section “Materials and Methods”) and the number of electrodes participating in the detected population bursts. Population bursts were detected in nine (four hippocampal and five cortical slices) of the 12 slices, with the number of population bursts being 45 ± 29.8 during *aCSF*, 190 ± 76.3 during *hCSF*, and 33 ± 23.1 during *washout*, which were significantly different (*p* = 0.0148). Using Dunn’s multiple comparison test, we found that the decrease in the number of population bursts comparing *hCSF* and *washout* was significantly different (*p* = 0.0201), but the number of population bursts was not significantly different between *aCSF* and *hCSF* (*p* = 0.3765) as well as *aCSF* and *washout* (*p* = 0.7158) (Figure 3A). In addition, we compared the relative change in population burst rate in these nine slices (see section “Materials and Methods” for definition of relative change). We found an average increase in the population burst rate from aCSF to hCSF of 52 ± 17% (*p* = 0.02, *t*-test), which decreased by 71 ± 16% (*p* = 0.002) when switching back from hCSF to aCSF.

The number of electrodes active in these population bursts was 22 ± 5.9 during *aCSF*, 84 ± 18.6 during *hCSF*, and 32 ± 18.1 during *washout*. When testing the three conditions with the paired, non-parametric Friedman test, a significant difference was found (*p* < 0.0001), and Dunn’s multiple comparison test found the increase in number of active electrodes during population bursts seen when comparing *aCSF* and *hCSF* significant (*p* = 0.0286) as well as the decrease between the *hCSF* and the *washout* (*p* = 0.0005), but the *aCSF* compared to the *washout* (*p* = 0.7158) was found non-significant (Figure 3A). Looking at individual slices (Figure 3A, individual points in the graph), it becomes evident that the hippocampal slices (circles) did not show the same clear increase in number of population bursts during hCSF as the cortical slices (triangles). In two of the hippocampal slices, we observed a decrease in the number of population bursts, when hCSF was washed in. In the cortical slices, an increase in population burst rate was recorded in all slices. In the hippocampal slices, we observed that the electrodes that were not active in aCSF often started to show spiking activity that seemed unrelated to the activity in the electrodes already active in aCSF (Figure 3B, hippocampal slice 5), and in other cases, these areas became active as a smaller network and had separated population bursts (Figure 3C, hippocampal slice 1). In the cortex slices, there was a clear shift toward higher level of synchronization in the network. In two cortical slices, the number of active electrodes was only slightly changing in response to the wash in of hCSF (less than 10%, slice 4, heat map in Figure 1D), but the activity recorded was highly synchronized and showed a high number of population bursts with an increased number of active electrodes/population burst (Figure 3D, cortical slice 4). Even in the one slice in which the number of active electrodes decreased in *hCSF*, cortex slice 3 (heat map in Figure 1D), the population bursts appeared and most of the activity in the slice was synchronized during *hCSF* (Figure 3E).

Lastly, we tested if aCSF with a high potassium concentration (6–8 mM, “high K” condition) could mimic the activity recorded in hCSF. The number of active electrodes and the number of burst identified on single electrodes did not change in the two conditions. The average spike rate showed a relative increase of 31% (*n* = 7, ^∗^ *p* < 0.05) in high potassium. The significant change, however, was due to one recording (Supplementary Figure S2A). The number of population bursts was different in the two conditions, but with strong variability across slices. In four slices, we detected an increasing population burst rate in high K when compared to hCSF, while in two slices, there was a decrease of the population burst rate (Supplementary Figure S2A). We present qualitative raster plots, which exemplify the different nature of activity (Supplementary Figure S2B); however, further experiments and more refined physiological readouts are required to identify the differences between the two conditions.

## Discussion

The activity of *in vitro* neuronal networks is highly dependent on the recording medium and is commonly experimentally measured in standard media or aCSF. In several recent studies, the activity in rodent cells and networks was compared between aCSF and hCSF and in general a significant increase in the activity was detected in all these studies (Bjorefeldt et al., 2015, 2019; Perez-Alcazar et al., 2016; Koch et al., 2019). In line with these studies, we found that the overall activity levels at the network (determined by 256 MEA recordings) and on the single-cell level (determined by CMOS-MEA and whole cell patch-clamp recordings) increased in human hippocampal and neocortical slices, when the slices were perfused with hCSF compared to aCSF.

### Human CSF Causes Changes in Single Cell and Network Activity

Using a 256-MEA, we observed an increase in activity levels by analyzing the number of active electrodes, spike rate, number of bursts, population bursts, and number of electrodes active in population bursts in hCSF compared to aCSF. This indicates that this phenomenon, previously observed only in animal tissue, is also preserved in human neurons and networks. The response when exposed to hCSF was rapid (present within minutes) and robust (lasting for the experimental protocol of 1 h). In a first step, we averaged these parameters for each slice recorded, and on average, the wash in of hCSF induced a significant increase in the number of active channels, spike rate, and number of busts followed by a fast decrease back to baseline when hCSF was washed out and replaced by aCSF. As a previous study demonstrated in rodent tissue, the general increase in activity was not explained by increased concentrations of potassium or other ions in the hCSF compared to aCSF (Bjorefeldt et al., 2015). Similarly, in our hCSF samples, we verified that the potassium concentration was in the same range as that in the aCSF. The number of active electrodes and increased spike rates might simply have been due to the fact that more cells depolarized and thereby had a higher probability of generating action potentials. This is in part supported by the data from a small set of whole cell patch-clamp recordings that showed in five out of six recorded cells an increase of spontaneous spiking of the cells combined with a significant increase of the membrane potential. However, we also observed a significant increase of burst frequency, number of population bursts, and number of active electrodes in a population burst, which indicated a network-driven increase of the activity levels in the presence of hCSF. While bursts in single electrodes might reflect an increase and induction of intrinsic bursting properties observed in rodent and human cortical pyramidal cells (Llinás and Steriade, 2006; Martell et al., 2010; Jacob et al., 2012), it could also reflect synaptically determined activity. To test this, we determined the presence and number of network-evoked population bursts. In the majority of the slices, such population bursts could be detected in aCSF and hCSF (nine out of 12 slices), which is in line with our previous work in human slice cultures (Schwarz et al., 2017). In hCSF, we found a significant increase in the number of population bursts and of the active electrodes within the population bursts compared to aCSF. These findings clearly indicate a surge of network function in the presence of hCSF compared to aCSF. This effect might be in principle due to several processes, including higher synchrony of the active elements in the network or a recruitment of cells into the network without an increase of the synchrony (van Drongelen et al., 2003; Suresh et al., 2016). Interestingly, the increase of activity was detected in both human cortical and hippocampal slices and seems to reflect a general phenomenon in distinct brain areas and also over different species (Bjorefeldt et al., 2016; Koch et al., 2019). The cortical slices showed a clear shift from random spiking to very synchronized burst activity detected as numerous population bursts and exemplified in Figure 3D. In hippocampal slices, the shift in synchronization was not as clear as in the cortical slices, but nevertheless showed an increase with more population bursts (Figure 3B).

### Potential Mechanisms of hCSF-Induced Activity Increase

As mentioned above, the increase of the activity in the presence of hCSF was most likely not induced by an alteration of the ionic composition. However, hCSF is in addition composed of a broad spectrum of neuromodulatory substances such as amino acids, peptides, and lipids (Bjorefeldt et al., 2018). All of these might participate in the modulation of activity levels and might even be dynamically influencing the activity of specific brain areas leading to changes in behavior. One fascinating example of such a modulation was observed in the study of sleep-deprived goats. From the CSF of the sleep-deprived animals, a specific factor could be isolated and transferred into other animals. This transfer caused a drastic increase in the slow wave sleep in these non-sleep-deprived animals (Pappenheimer et al., 1975). Subsequently, this substance was identified to be a muramyl peptide (Krueger et al., 1982). Obviously, this pathway of CSF-mediated modulation of neuronal activity levels is not limited to physiological processes, but can also be observed in pathological situations and lead to very specific changes in neuronal behavior, which is linked to the presence of specific components such as NMDA receptor antibodies (Dalmau et al., 2008; Kreye et al., 2016). Since the hCSF for our study was collected from patients in an anonymous way, we cannot exclude the possibility that the increase of the activity levels is due to the presence of medications that the patients received. We used hCSF that had to be collected from patients for therapeutic reasons but did not show pathological properties in the basic biochemical composition. We pooled the hCSF samples from many patients and so subtle differences or effects by medications are probably not detectable at this time and were not the aim of this study. Also, we believe that the increase of activity we describe in our study is most likely a general phenomenon and should be considered as a basic difference to aCSF that lacks many major components of the hCSF. Arguments for this are the general overall increase observed in two distinct areas of the brain (cortex and hippocampus) and the results of previous studies in distinct species, showing all in general an increase of the neuronal activity (Bjorefeldt et al., 2018). While there might be distinct actions of several components, also reducing neuronal activity levels, we believe that the sum effect of hCSF versus aCSF leads to increasing the activity of the network probably by several stimulatory effects.

### Specific Activation and Inactivation of Subpopulations of Neuronal Ensembles

Interestingly, the general and overall increase of the activity in the measured slices was not true for all parts of the network, and we found also several areas and single cells that decreased their activity levels in hCSF or were only active in aCSF. This finding indicates a more complex interplay of the network components induced by the switch to hCSF and a reconfiguration of the network with specific activation and inactivation of subpopulations of neuronal ensembles. One hypothesis to the specific activation and inactivation of different neuronal ensembles could be that an increased excitability among fast spiking interneurons, previously reported in rodent studies (Bjorefeldt et al., 2019), will in turn inhibit other neuron populations that would turn silent in hCSF. By characterizing the cell-specific waveform of the extracellularly recorded spikes and combining this with the spike rate of the same cell, it may be possible to discriminate between interneurons and excitatory neurons (Csicsvari et al., 1999). Interneurons recorded *in vivo* have been characterized by narrower waveforms, higher spike rate, and distinct bursting behavior compared to principal neurons (Barthó et al., 2004; Viskontas et al., 2007; Reyes-Puerta et al., 2015). As hCSF did not induce a change in the somatic waveform compared to the waveform recorded in aCSF, we analyzed if the waveform width could be used to search for cell-type specificity. However, even if the five cells identified in hCSF with narrow width would be fast spiking interneurons, the majority of cells (*n* = 49) identified only in hCSF had indistinguishable waveforms from the average. Thus, we cannot conclude any cell-type-specific activation by hCSF. Future work will reveal if in brain slices electrophysiological parameters are able to separate neuronal populations, which seems impossible in cell cultures (Weir et al., 2015), but may be aided by additional information extracted from the high-density electrode array (Jia et al., 2019).

Furthermore, we performed intracellular recordings in a small set of cortical neurons and found in all cells a direct depolarization of the membrane potential and induced firing in five out of six of the recorded neurons. This is in line with previous studies that showed similar responses in rodent brain slices of the membrane potential and the firing of neurons (Bjorefeldt et al., 2016). However, several other mechanisms, such as changes in excitatory and inhibitory synaptic transmission, are likely to participate to the alterations of activity observed on the network level. This is supported by the aCSF experiments with elevated potassium that showed increased firing, but a distinct pattern revealed by the 256-MEA recordings (Supplementary Figure S2).

Differences in calcium concentration have recently been shown between aCSF and hCSF followed by higher rates of spontaneous spikes recorded in rodent slices if the calcium concentration is not increased to aCSF-level (Forsberg et al., 2019). This could be a possible explanation for the increase in excitability we have observed in the human tissue slices, but future experiments specifically designed to test this will be needed before any conclusions can be made.

### Potential Significance of Altered Activity in hCSF

In a recent study, hCSF induced an increase in spontaneous gamma oscillations in CA1 and CA3 area of mouse hippocampal slices and the increase was shown to rely on muscarinic acetylcholine receptors (Bjorefeldt et al., 2019). The increase in bursts and population bursts induced by hCSF observed in our human tissue slices may indicate that the neurons usually engaged in oscillatory activity such as gamma oscillations more readily do so spontaneously when hCSF is present. In addition, in a small set of slices, we directly tested if aCSF with increased potassium levels could mimic the effect of hCSF. Interestingly, the activity (active electrodes, and spike rate) did also increase in high potassium aCSF compared to aCSF with physiological potassium levels. But there was less indication for synchrony measured by total number of population bursts vs number of single channel bursts and the total number of active electrodes involved in the population burst. A higher number of measurements and also single cell and synaptic measurement will be needed in future studies to determine similarities and differences between high potassium and hCSF-induced activity levels.

### Human Tissue, Human Slice Cultures, and hCSF as Experimental Models

The resected human tissue is of great importance for translational research, especially for frequent neurological diseases such as epilepsy. The tissue provides a unique possibility to understand the physiological as well as pathological mechanisms in the human neuronal network circumventing the problem of poor translatability from animal models. Several studies using human tissue have already moved the research field forward, providing new insights into pathological mechanisms underlying temporal lobe epilepsy (Beck et al., 2000), thereby not only helping to develop new antiepileptic drugs (Straub et al., 2001), but also increasing the general physiological understanding of cortical activity (Verhoog et al., 2013; Szegedi et al., 2017; Kalmbach et al., 2018).

Due to the limited access to resected tissue, it is critical that results and studies can be compared between laboratories and different research groups working with this valuable tissue. Using standardized methods and solutions is a key factor for enabling such comparisons; as recently pointed out by de la Prida and Huberfeld (2019), aCSF is as such a standardized solution. The results of the present study and others (Perez-Alcazar et al., 2016; Bjorefeldt et al., 2018; Forsberg et al., 2018) show a clear difference in the neuronal firing behavior when comparing aCSF and hCSF. To understand mechanisms during both physiological and pathological activity within the human brain network, it is crucial to mimic the environment in the brain as closely as experimentally possible.

## Conclusion

Using hCSF instead of aCSF, when recording from human tissue, resulted in significant changes of the single-cell and network activity. We demonstrated that the changes are complex and that the specific activation and inactivation of certain neurons cannot simply be explained by different effects on excitatory or inhibitory neurons. In the present study, we have only revealed the major changes and highlighted the complexity, scratching the surface of the interesting effects hCSF has on individual neurons and neuronal networks.

## Data Availability Statement

All datasets generated for this study are included in the article/Supplementary Material.

## Ethics Statement

The studies involving human participants were reviewed and approved by the Ethics Committee of the University of Tübingen, Germany (Approval Nos. 338/2014BO2 and 338/2016A). The patients/participants provided their written informed consent to participate in this study.

## Author Contributions

JW and AC performed microelectrode array recordings. NS, BU, NL, JH, TW, HK, and JW did tissue preparation and culturing. BU, NL, and HK did patch-clamp recording. JW, AC, and GZ did data analysis and writing. All authors contributed to proofreading and study design.

## Conflict of Interest

The authors declare that the research was conducted in the absence of any commercial or financial relationships that could be construed as a potential conflict of interest.

## Supplementary Material

FIGURE S1 Intracellular whole cell patch clamp recordings reveal depolarization of the resting membrane potential (RMP). **(A)** Exemplary patch-clamp recording of a cortical neuron during the transition from aCSF to hCSF (“hCSF wash in”) and from hCSF to aCSF (“aCSF wash in”). **(B)** During hCSF, the RMP depolarized significantly (^∗^*p* < 0.05) from −73.9 to −67.3 mV, which was reversed by washout with aCSF. **(C)** Reconstruction of a recorded excitatory cortical neuron.

FIGURE S2 Comparison of activity in aCSF with high K to the activity in hCSF. **(A)** The general activity measured by the number of active electrodes, spike rate, number of single bursts and average durations, and number of populations bursts (in a time window of 30 min) in the two conditions hCSF (red) and aCSF with high K (black symbols). Data from hippocampal slices are marked by circles, while cortical slices are marked by triangles. **(B)** Representation of different activity patterns under hCSF (left) and aCSF with high K (right). **(Bi)** Upper panels: Summarized spike rate trace from hippocampus slice 2 (see heat map in Figure 1D) in hCSF and in aCSF with high K in a 10-s moving window. Lower panels: Raster plots showing the activity on all 252 recording channels. Activity is normalized to the activity in aCSF (see Figure 1D). Representation of activity in hippocampal slice 3 **(Bii)**, in cortical slice 1 **(Biii)**, and in cortical slice 4 **(Biv)**. Differences in the spatial activity patterns in the two conditions are visible.
