# Supplementary figures and images for: Human Cerebrospinal Fluid Induces Neuronal Excitability Changes in Resected Human Neocortical and Hippocampal Brain Slices
Source: Front Neurosci. 2020 Apr 21;14:283. doi: 10.3389/fnins.2020.00283 (PMC7186381; doi:10.3389/fnins.2020.00283)

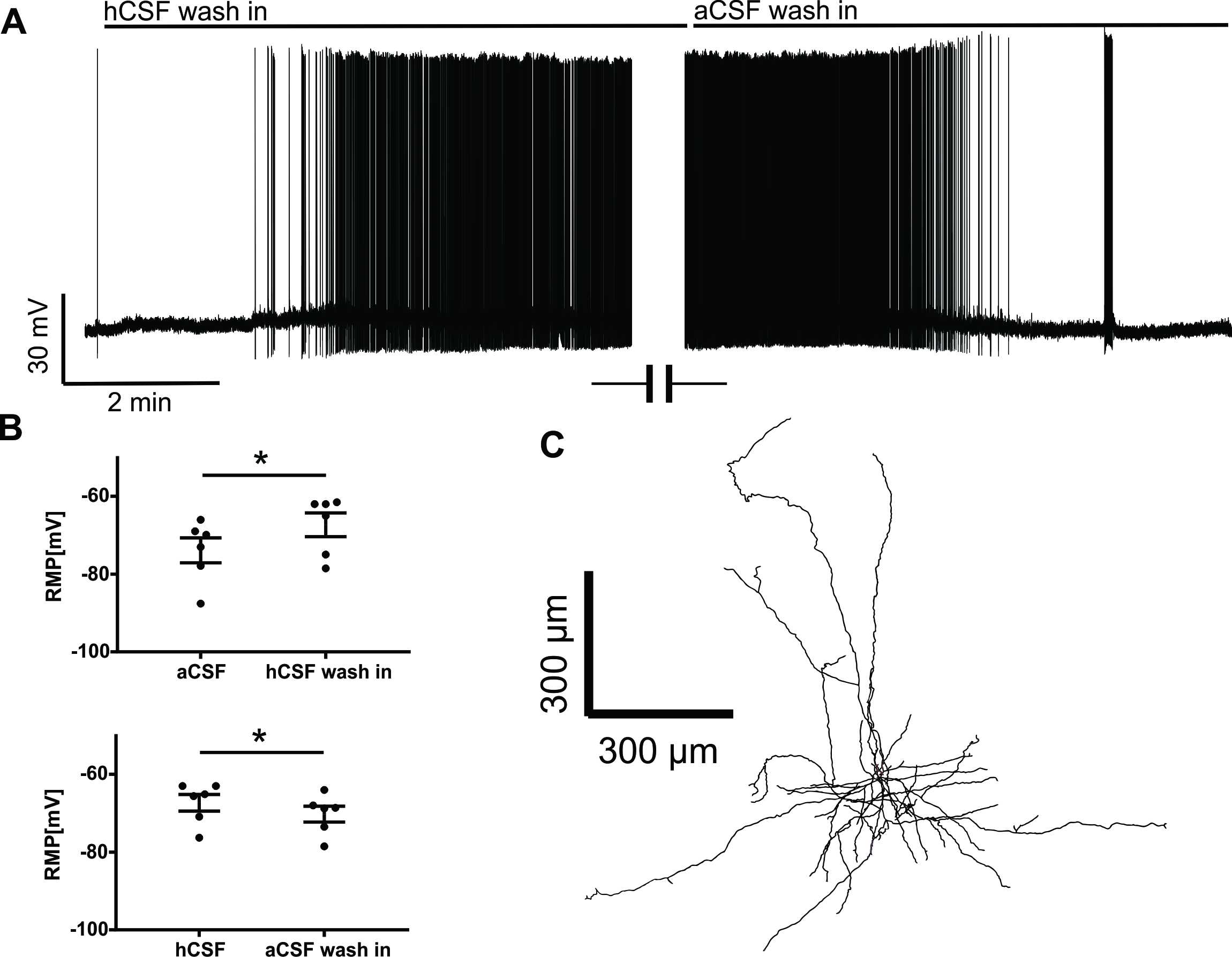

Supplement: FIGURE S1 — Intracellular whole cell patch clamp recordings reveal depolarization of the resting membrane potential (RMP). (A) Exemplary patch-clamp recording of a cortical neuron during the transition from aCSF to hCSF (“hCSF wash in”) and from hCSF to aCSF (“aCSF wash in”). (B) During hCSF, the RMP depolarized significantly (∗p < 0.05) from −73.9 to −67.3 mV, which was reversed by washout with aCSF. (C) Reconstruction of a recorded excitatory cortical neuron. [file Image_1.jpg]

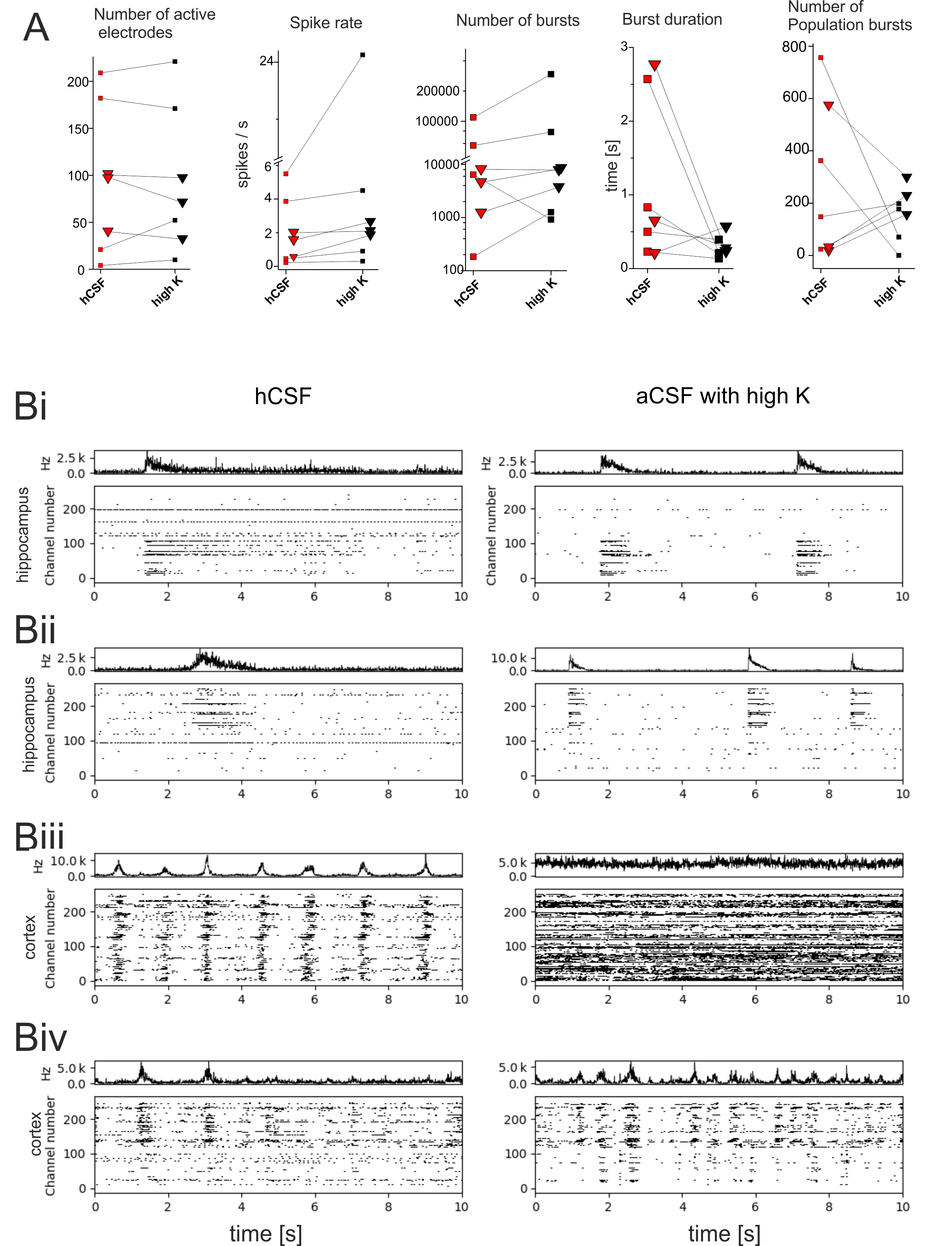

Supplement: FIGURE S2 — Comparison of activity in aCSF with high K to the activity in hCSF. (A) The general activity measured by the number of active electrodes, spike rate, number of single bursts and average durations, and number of populations bursts (in a time window of 30 min) in the two conditions hCSF (red) and aCSF with high K (black symbols). Data from hippocampal slices are marked by circles, while cortical slices are marked by triangles. (B) Representation of different activity patterns under hCSF (left) and aCSF with high K (right). (Bi) Upper panels: Summarized spike rate trace from hippocampus slice 2 (see heat map in Figure 1D) in hCSF and in aCSF with high K in a 10-s moving window. Lower panels: Raster plots showing the activity on all 252 recording channels. Activity is normalized to the activity in aCSF (see Figure 1D). Representation of activity in hippocampal slice 3 (Bii), in cortical slice 1 (Biii), and in cortical slice 4 (Biv). Differences in the spatial activity patterns in the two conditions are visible. [file Image_2.png]
